# A Novel Mutation in the FYCO1 Gene Causing Congenital Cataract: Case Study of a Chinese Family

**DOI:** 10.1155/2022/5838104

**Published:** 2022-08-26

**Authors:** Shuping Mei, Jingwei Lin, Zhen Liu, Cheng Li

**Affiliations:** ^1^Department of Ophthalmology, Xiang'an Hospital of Xiamen University, Eye Institute & Affiliated Xiamen Eye Center, School of Medicine, Xiamen University, Xiamen 361102, China; ^2^Fujian Provincial Key Laboratory of Ophthalmology and Visual Science, School of Medicine, Xiamen University, Xiamen 361102, China

## Abstract

Congenital cataract is the most important global cause of visual impairment in children. Autosomal dominant and autosomal recessive inheritance account for the majority of the hereditary nonsyndromic congenital cataract. The function of FYCO1 gene is to guide the transport of the microtubule-directed vesicles. Mutations in the FYCO1 gene may cause cataracts. We reported a novel nonsense mutation in FYCO1 (c.1411C > T, P. R471 ∗), which could cause nonsyndrome autosomal recessive congenital cataract. We underwent an ophthalmology examination of all participants and collected blood samples from all participants and extracted genomic DNAs. By whole exome sequencing, we found that this family carried an unreported mutation in the FYCO1 gene: c.1411C > T, P. R471 ∗. Sanger sequencing was performed to verify the mutation. We used ITASSER and PYMOL to predict and compare the structure and function of the mutated proteins. Using SIFT software and referring to the relevant guidelines of ACMG, the mutation was determined to be pathogenic. The models suggested that the nonsense mutation p.R471∗ resulted in a profound disruption of the FYCO1 protein structure. This report expands the locus information of the FYCO1 mutations.

## 1. Introduction

Lens is one of the important refractive stroma in the eyeball [1]. The newborn lens fiber cells express the key proteins needed to realize the structure and transparency of the mature lens and completely eliminate the nucleus and all organelles, in order to ensure the normal function of the lens [2, 3]. Cataract is characterized by an opacity and protein degeneration of the lens. Human crystallin has three major protein components, including *α*-crystallin, *β*-crystallin, and *γ*-crystallin. Among them, the most important crystallin is *α*-crystallin, which is composed of A and B subunits [4–6]. A common feature of several cataracts is the misfolding of crystallin [7]. Thus, misfolding of crystallin can be an important cause of cataract [8].

Congenital cataract (CC) is the most important global cause of visual impairment in children. According to statistical analysis, about 1/4^th^ of CCs are caused by genetic defects [9]. In the early stage of the fetal development, the disorder of protein metabolism in the lens affects the transparency of the embryonic lens and leads to the occurrence of CC. The increase of protein content in the lens worsens the microstructure of the lens and leads to the opacity of the lens [10]. To date, more than 50 different sites of CC have been identified [11]. Autosomal dominant and autosomal recessive inheritance account for the majority of the hereditary nonsyndromic CCs. X-linked recessive inheritance accounts for only a small fraction [12]. Nonsyndromic CC occurs in 1 to 6 out of every 10000 live births [13], of which about 1/3^rd^ of the CC cases are familial [14]. In addition, about 70% of the cases are nonsyndromic and can only lead to lens damage [15]. The gene associated with nonsyndrome autosomal recessive CC (ARCC) includes EphA2 (gene ID1969, OMIM 176946) [16], GJA8 (gene ID2703, OMIM 600897) [17], FOXE3 (gene ID2301, OMIM 601094) [18], FYCO1 (gene ID 79443, OMIM 607182) [11, 19–21], GCNT2 (gene ID2651, OMIM 600429) [22], AGK (gene ID 55750, OMIM 610345) [23], DNMBP (gene ID 23268, OMIM 611282) [24], and CRYBB1 (gene ID 1414, OMIM 600929) [25]. Numerous mutations have been reported in the FYCO1 gene, which result in loss of function of FYCO1, resulting in ARCC [19, 26].

In the present study, we report a novel mutation in the FYCO1 gene leading to congenital cataract in a Chinese family, using whole exon sequencing. Furthermore, the clinical features and treatment process of the case are described in detail.

## 2. Subjects and Methods

### 2.1. Subjects

**Figure 1:**
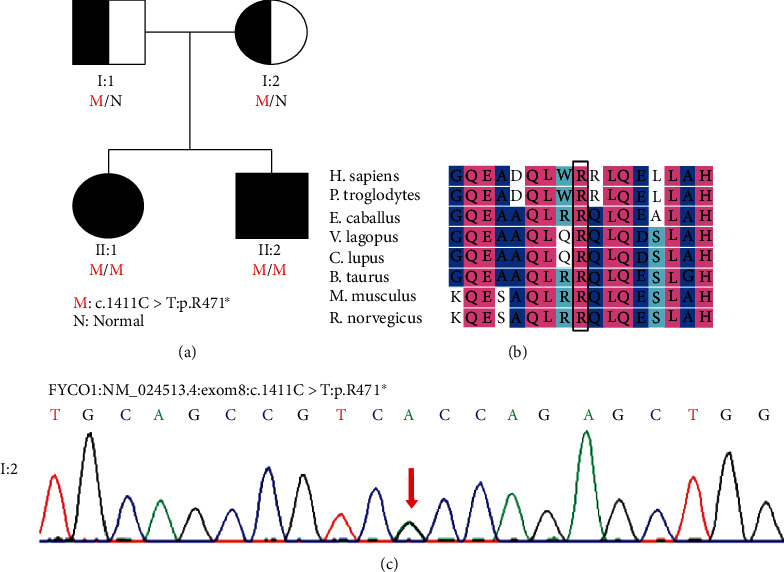
Family history and genomic analysis. (a) Detailed family history. The daughter and son with congenital cataracts were homozygous mutations (c.1411C > T: p.R471∗). The parents carried heterozygous mutations without any symptoms. (b) Evolutionary conservation of the altered amino acid residues. (c) Sanger sequencing of the mother's DNA. Mutation validation results showed that the sequence on the template chain: G was replaced by A. This is equivalent to C on the coding chain being replaced by T.

**Figure 2:**
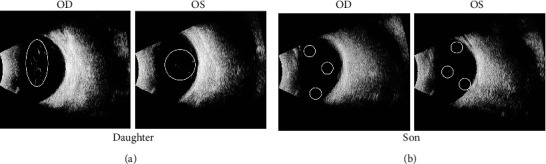
Ocular ultrasound images. (a) Vitreous bodies with mild opacity of the daughter. (b) Vitreous bodies with mild opacity of the son. The daughter's vitreous opacity was more pronounced and none of them found the lens.

The subject for the present study included a Chinese family with CC. The family consisted of two children with CC and their parents whose ocular presentations were normal (Figure 1(a)). The daughter was 18 years old. When she was 1 year old, she underwent a “binocular cataract extraction” because of “binocular congenital cataract.” She was regularly reviewed after the operation, and she had not been implanted with intraocular lens and wore glasses for a long time. In 2021, after detailed ophthalmological examination and evaluation in the Xiang'an Hospital affiliated to Xiamen University, secondary intraocular lens implantation was performed in the left eye and right eye, respectively. The son was 10 years old, and his symptoms were the same as his sister. At the age of 1, he underwent a “binocular cataract extraction” because of “binocular congenital cataract.” He was regularly reviewed after the operation, but had not been implanted with intraocular lens and wore glasses for a long time. In 2021, after detailed ophthalmological examination and evaluation in Xiang'an Hospital affiliated to Xiamen University, secondary intraocular lens implantation was performed in the left eye and right eye, respectively. The results of clinical examination and evaluation of the two patients are detailed in the table below (Table 1). The vitreous bodies of the two children presented a mild opacity (Figure 2). The parents' eye manifestations were normal, and there was no cataract or any other disease. Prior to the genetic testing, all participants voluntarily signed an informed consent form. This study was permitted by the Ethics Committee of Xiang'an Hospital of Xiamen University and was in line with the Helsinki Declaration.

### 2.2. Methods

#### 2.2.1. Extraction of DNA

Blood samples were collected from the subjects after obtaining their informed consent. Total DNA extraction from blood was performed using the TIANamp Blood DNA kit (Tiangen Biotech Co., Ltd., China). The integrity of 2% agarose gel electrophoresis showed that the main band of DNA was obvious, and there was no degradation. Using NanoDrop1000 spectrophotometer (Thermo Fisher, USA) to detect the purity and concentration, the concentration of OD260/280 was 18-20, the concentration was greater than 20 ng/ul, and the total amount was greater than 500 ng.

#### 2.2.2. FYCO1 Mutation Detection

In order to study the cause of the disease, we performed a whole exome sequencing (WES) on the daughter, son, and the father. Hieff NGS OnePot DNA Library Prep Kit (Yeasen Biotech Co., Ltd., Shanghai, China) was used for DNA enzyme fragmentation and whole genome library construction. The whole exon group library of the parents and their parents was constructed by hybridizing and capturing the whole exon group library with the Human Comprehensive Exome Panel (Twist Bioscience Co., Ltd., USA). The PE100 mode was used for double-terminal sequencing on the MGISEQ-T7 sequencing platform.

The BWA software was used to compare the hg19 version of the human genome reference sequence provided by UCSC, and the SNV and InDel mutations were found through the HaplotypeCaller of GATK. The pathogenicity was evaluated using the SIFT software (http://siftdna.org), and the variation was determined to be a pathogenic variation by referring to the relevant guidelines of the ACMG.

Sanger sequencing was performed on the sample obtained from the mother to confirm the pathogenic variant in the patients. Two primers (forward primer: GCCTCTTGCAGACTGGAGTT; reverse primer: ATGCAGGAGCTAGGGGAGAA) were used to verify the region of the gene mutation. Sanger sequencing was performed according to the standard protocol. PCR was performed by placing each 20 ng of human DNA in standard PCR buffer (50 *μ*l). The PCR product was then purified using the Cwbio gel extraction kit (Cwbio, Beijing, China). Finally, an ABI 3500 Genetic Analyzer (Applied Biosystems, Foster City, CA) was used to sequence the DNA.

## 3. Results

### 3.1. Mutational Analysis

By WES, we found that this family carried an unreported mutation in the FYCO1 gene: c.1411C > T, P. R471 ∗. The daughter and the son with CC exhibited homozygous mutations, while the normal parents exhibited heterozygous mutations. We also verified this mutation in FYCO1 by Sanger sequencing of the mother's DNA (c.1411C > T, p. R471∗) (Figure 1(c)). In addition, the aforesaid mutation was not found in the 1000Genome Project database and the GnomAD exon database.

The original amino acid (p.Arg471) of the mutated region was found to be well conserved. Using SIFT software (http://sift-dna.org) and referring to the relevant guidelines of ACMG, the mutation was determined to be pathogenic. From this point of view, the observed FYCO1 mutation (c.1411C > T, p. R471 ∗) is highly likely to be a pathogenic mutation.

### 3.2. Protein Structures

**Figure 3:**
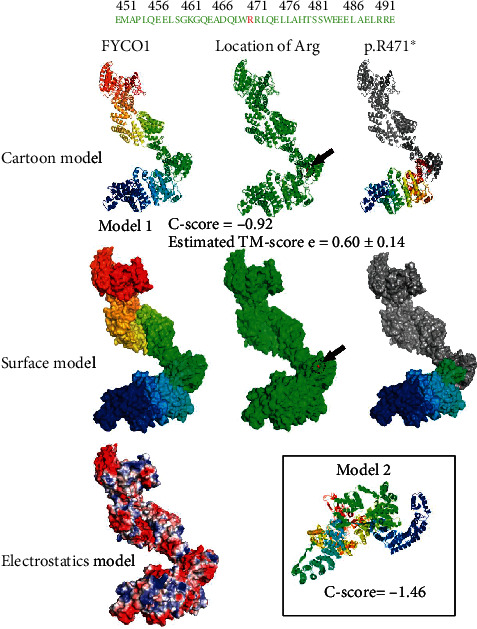
3D-structure model of the variant (The FYCO1 protein structure is shown in different color. The location of the mutation p.R471∗ is marked in red in the green color scheme. The protein structure disruption caused by the nonsense mutation p.R471∗ is shown in gray. The models are predicted by I-TASSER (include model 1 and model 2). C-score is typically in the range of [-5, 2], where a C-score of a higher value signifies a model with a higher confidence. A TM − score > 0.5 indicates a model of correct topology (model 1 C − score = −0.92; model 2 C − score = −1.46; model 1 TM − score = 0.60 ± 0.14)).

To investigate the potential harm of this FYCO1 protein mutation, we modeled this variant using some software. We use ITASSER (https://zhanggroup.org/I-TASSER/) [27–29] and PYMOL (https://pymol.org/2/) to predict and compare the structure and function of the mutated proteins. The models suggested that the nonsense mutation p.R471∗ resulted in a profound disruption of the protein structure (Figure 3).

### 3.3. Clinical Treatment

The daughter was 18 years old in the course of this study. When she was 1 years old, she underwent a “binocular cataract extraction” because of “binocular congenital cataract.” She was regularly reviewed after the operation, and she had not been implanted with intraocular lens and wore glasses for a long time. In 2021, after detailed ophthalmological examination and evaluation at the Xiang'an Hospital affiliated to the Xiamen University, secondary intraocular lens implantation was performed in the left eye and the right eye, respectively, and the postoperative visual acuity recovered well. Her brother was 10 years old in course of the study, and his symptoms were the same as his sister. At the age of 1, he underwent a “binocular cataract extraction” because of “binocular congenital cataract.” After regular follow-up, he was not implanted with an intraocular lens and wore glasses for a long time. In 2021, after detailed ophthalmological examination and evaluation at the Xiang'an Hospital, secondary intraocular lens implantation was performed in the left eye and the right eye, respectively, and the postoperative visual acuity recovered well.

## 4. Conclusions

FYCO1 has a PI (3) P-binding protein and a Rab7 effector protein. Studies have shown that its function is to guide the transport of the microtubule-directed vesicles. And immunofluorescence staining shows that FYCO1 is localized to autophagosomes [30]. The longest region in the structure of FYCO1 is the coiled-coil region, where most of the mutations occur. In one side of the coiled-coil region lies the RUN domain and three regions in the other side, including FYVE, LIR, and GOLD [31].

After the formation of autophagosome, the adventitia of autophagosome fuses immediately with the lysosome to form autolysosome. Rab7 plays an important role in this process [32, 33]. Rab7 recruits FYCO1 in the formation of the autophagosome [34]. As Rab7 is bound to GTP, FYCO1 preferentially interacts with it [19]. FYCO1 has also been shown to bind to the outer membrane of the autophagosome through its FYVE domain. Some studies have proposed that autophagosomes are connected to the microtubule motor proteins through FYCO1, enabling a smooth autophagosome transport. Deletion of the FYCO1 leads to autophagosome aggregation [26].

Some other researchers believe that FYCO1 directly interacts with *α* A-and *α* B-crystallin, which are the main components of the lens. Using FYCO1 knockout mice, it was found that increased *α*A- and *α*B-crystallin precipitates in the lenses. The findings suggest that normal FYCO1 function may be linked to the autolysosomes, wherein the damaged *α* crystal proteins are transported and degraded. Therefore, when FYCO1 is mutated, it cannot perform its normal function, and the damaged *α*-crystallin cannot be degraded, which eventually leads to the cataract [21]. From this point of view, the function of FYCO1 is very important, as its mutation causes cataract, thereby affecting people's lives.

In this study, both the daughter and son of this family suffered from CC and underwent “binocular cataract extraction” at the age of one year. They were reexamined regularly after operation, but no intraocular lens was implanted and wore glasses for a long time. Later, a two-stage intraocular lens implantation was performed in the left eye and the right eye, and the postoperative visual acuity was improved. The lens and other eye tissue functions of their parents were normal. Therefore, we speculate that the CC in this family may be a nonsyndromic autosomal recessive disease. After sequencing the whole exon group of the family, we found a new FYCO1 gene mutation (c.1411C > T, p. R471 ∗). It resulted in the mutation of the arginine in the coiled-coil domain to a stop codon, resulting in a disturbing change in the protein structure of FYCO1. The parents of the patients carried heterozygous mutations without any symptoms, while the two children with CCs were homozygous mutations. This result is consistent with our conjecture.

Our study proves that FYCO1 is closely related to the occurrence of CC. This report expands the locus information of the FYCO1 mutations and can be helpful in an accurate diagnosis of CC, so as to better prevent and treat it.

## Figures and Tables

**Table 1:** Specialist examination of two patients.

Patient	Daughter		Son	
	OD	OS	OD	OS
Age (years)	18		11	
Age when the lens removed (years)	1		1	
Correct vision	0.3	0.8	0.15	0.5
Intraocular pressure (mmHg)	17	17	15	18
Pupil position	Up	Centre	Centre	Centre
Pupil shape	Not round	Round	Round	Round
Pupillary diameter (mm)	2	3	3	3
Reflection on light	Dullness	Dullness	Dullness	Dullness
Corneal endothelial cells (/mm^2^)	3984	3471	3056	3156
Lens	Lacking	Lacking	Lacking	Lacking
Retina	Normal	Normal	Normal	Normal
Vitreous body	Mild opacity	Mild opacity	Mild opacity	Mild opacity
Eyeball	Normal	Normal	Horizontal tremor	Horizontal tremor
Eye axis (mm)	25.05	22.88	23.52	22.86

## Data Availability

The data that support the findings of this study are available on request from the corresponding author, C.L.
